# In Vitro Anticancer MCF-7, Anti-Inflammatory, and Brine Shrimp Lethality Assay (BSLA) and GC-MS Analysis of Whole Plant Butanol Fraction of* Rheum ribes* (WBFRR)

**DOI:** 10.1155/2019/3264846

**Published:** 2019-06-23

**Authors:** Jahangir Khan Achakzai, Muhammad Anwar Panezai, Muhammad Ayub Kakar, Abdul Manan Kakar, Shahabuddin Kakar, Javed Khan, Nazima Yousaf Khan, Inayatullah Khilji, Ajab Khan Tareen

**Affiliations:** ^1^Institute of Biochemistry, University of Balochistan, Quetta 87300, Pakistan; ^2^Department of Zoology, University of Balochistan, Quetta 87300, Pakistan; ^3^Department of Microbiology, Quaid-i-Azam University, Islamabad 45320, Pakistan

## Abstract

In this study, GC-MS analysis has shown that whole plant butanol fraction of* rheum ribes* (WBFRR) comprises of 21 compounds which exhibited anticancer (MCF-7) activity having IC_50_ value of 36.01± 0.26. MTT assay (MCF-7), Oxidative Burst assay using chemiluminescence technique, and B-Hatching techniques were the methods used for anticancer MCF-7, anti-inflammatory, and Brine Shrimp Lethality Assay (BSLA). GC-MS was used for structural elucidation. Whole plant methanol extract of* rheum ribes* (WMERR), whole plant n-hexane fraction of* rheum ribes* (WHFRR), and whole plant aqueous fraction of* rheum ribes* (WAFRR) were inactive against anticancer (MCF-7) cell line. Whole plant methanol extract of* rheum ribes* (WMERR), whole plant aqueous fraction of* rheum ribes* (WAFRR) and whole plant butanol fraction of* rheum ribes* (WBFRR) showed anti-inflammatory activity on ROS having IC_50_ value of 23.2±1.9, 24.2±2.7 and 12.0±0.6. Whole plant butanol fraction of* rheum ribes* (WBFRR) showed Brine Shrimp Lethality with LD50 693.302 while whole plant methanol extract of* rheum ribes* (WMERR) and whole plant aqueous fraction of* rheum ribes* (WAFRR) showed high lethality at highest concentration. This study revealed that whole plant butanol fraction of* rheum ribes* (WBFRR) exhibited significant anticancer (MCF-7) activity. In the near future, the constituent of whole plant butanol fraction of* rheum ribes* (WBFRR) can be the alternative drug against MCF-7 cell line with least toxicity and side effects.

## 1. Introduction

*Rheum ribes*, the subtropical and temperate regional plant, belongs to Polygonaceae family. The English name of this plant is rhubarb which is of three types, for instance, the Rhapontic rhubarb, the Himalayan or Indian rhubarb, and the Chinese rhubarb [1]. Plenty of active phytochemical and crude drugs are present in this plant of Asian countries [2]. The biologically active phytochemicals are flavonoids (quercetin 3-O-rutinoside, quercetin 3-O-rhamnoside, quercetin 3-O-galactoside, and catechin), anthocyanins (cyaniding 3-glucoside and cyaniding 3-rutinoside), stilbene (desoxyrhapontigenin and trans-rhapontigenin), and anthraquinones (emodin, physcion, aloe-emodin, chrysophanol, and rhein). Ethnomedicinal properties of* rheum ribes* are laxative, antioxidative, analgestic, antidiabetic, antimutagenic, ascathartic, anti-inflammatory, anticancer, antibacterial, hepatoprotective, antiplatelet, diarrhea, measles, cholagogue, stomachic, smallpox, hemorrhoids, antiemetic, and antipsoriatic [3–10]. In this research study, anticancer MCF-7, anti-inflammatory, and Brine Shrimp Lethality Assay (BSLA) and GC-MS analysis are analyzed.

## 2. Materials and Methods

### 2.1. Plant Material

### 2.2. Extraction

The plant* rheum ribes* was collected from the mountains of Chaman, Balochistan, Pakistan, and identified by a taxonomist Prof. Dr. Rasool Bakhsh Tareen, Department of Botany, University of Balochistan, Quetta, Pakistan. 3kg whole plant of* rheum ribes* was dried in shade and then grinded, soaked in 5 liter of methanol, kept for 7 days, and shaken daily. After a 7-day period, methanol containing whole plant of* rheum ribes* was filtered and vaporized with the help of rotary evaporator. Dried semisolid whole plant methanolic extract of* rheum ribes* (WMERR) was 48.6 gm. This crude extract 11.5gm was examined for anticancer MCF-7, anti-inflammatory, and Brine Shrimp Lethality Assay (BSLA) while the remaining extract was fractionated with solvents, for instance, n-hexane, aqueous, and butanol.

### 2.3. Fractionation of Crude Extract

In a separatory funnel, with crude extract two solvents were added such as aqueous and n-hexane. The separatory funnel was shaken thoroughly to create two layers such as aqueous layer and n-hexane layer. Both solvents were separately vaporized with the help of rotary evaporator to form whole plant n-hexane fraction of* rheum ribes* (WHFRR) 0.1 gm and whole plant aqueous fraction* of rheum ribes* (WAFRR) 23.6 gm. Both fractions were examined for anticancer MCF-7, anti-inflammatory, and Brine Shrimp Lethality Assay (BSLA) while whole plant aqueous fraction of* rheum ribes* (WAFRR) was further fractionated with a solvent such as butanol [11, 12].

### 2.4. Fractionation of Whole Plant Aqueous Fraction of* rheum ribes* (WAFRR)

Whole plant aqueous fraction of* rheum ribes* (WAFRR) was fractionated with solvent such as butanol.

### 2.5. Formation of Whole Plant Butanol Fraction of* rheum ribes* (WBFRR)

In a separatory funnel, with whole plant aqueous fraction of* rheum ribes* (WAFRR), two solvents were added such as butanol and aqueous. The separatory funnel was shaken thoroughly to create two layers such as butanol layer and aqueous layer. Both solvents were separately vaporized with the help of rotary evaporator to form whole plant butanol fraction of* rheum ribes* (WBFRR) 2.5gm and whole plant aqueous fraction of* rheum ribes* (LAFRR). Whole plant butanol fraction of* rheum ribes* (WBFRR) was examined for anticancer MCF-7, anti-inflammatory, and Brine Shrimp Lethality Assay (BSLA) [11, 12].

### 2.6. MTT Assay (MCF-7 Cell Lines)

In this assay, Dulbecco's Eagle modified medium, containing ten percent fetal bovine serum, was used for the culturing of MCF-7 cell lines which were then kept in five percent CO_2_ and incubated at 37°C. MCF-7 cells were collected when confluency was developed and 8000 cells per well were plated in 96 well flat. After 24 hours, the extracts and fractions with 50 ug/ml were added and then incubated for 48 hours. After incubation, extracts and fractions were removed. To each well, 0.5mg/ml MTT was added and kept in incubator for 3 hours at 37°C. MTT was reduced into Formazan crystals which was then dissolved in 100ul DMSO and at 570 nm absorbance was taken using micro-plate reader (Spectra Max plus, Molecular Devices, CA, USA). In this assay, doxorubicin was used as a standard drug. The decrease in viable cells or percent inhibition was calculated with the help of following formula:

% Inhibition = 100 - (mean of O.D. of test compound - mean of O.D. of negative control)/(mean of O.D. of positive control - mean of O.D. of negative control) x 100)

For the calculation of IC_50_ 20 mM stock solution of extracts and fractions was diluted into working solution with 50 uM and then in order to get less than 50 percent inhibition, working solution was further diluted in serial dilutions. With the help of EZ-fit5 software, IC50 was calculated [13].

### 2.7. Anti-Inflammatory Assay

*Oxidative Burst Assay Using Chemiluminescence Technique*. In this technique, 25 ul diluted whole blood HBSS^++^ and 25 ul of extracts and fractions were incubated for 15 min at 37°C and then plated in 96 well plates. Control wells contain HBSS^++^ and cells while blank wells contain HBSS^++^. 25 ul luminol and 25 ul serum opsonized zymosan were added into each well. In terms of relative light units, the level of ROS was recorded in luminometer. In this assay, Ibuprofen with IC_50_ 11.2 ± 1.9 is used as a standard drug [14].

### 2.8. Brine Shrimp Lethality Assay

*B-Hatching Techniques*. Scatter 50 mg of brine shrimp eggs in a hatching tray which was already half filled with filtered brine solution. Put it in incubator at 23°C. Take 20 mg of extracts and fraction and dissolve it in 2ml of solvent. Transfer 5, 50, and 500 ul from this solution to 3 vials. Leave them overnight to allow the solvent to evaporate. With the help of Pasteur pipette put 10 larvae per each vial. Add 5ml seawater. Under illumination, for 24 hours, incubate them at 25-27°C. For positive and negative controls, add reference cytotoxic drug along with solvent in other vials. For the determination of LD_50_, Finney computer program was used [15–17].

### 2.9. Gas Chromatography Mass Spectrometry (GC-MS) Analysis: Triple Quadrupole Acquisition Method MS Parameters

For identification and quantification of* rheum ribes *compounds, 2 ul of* rheum ribes* extract or fraction was directly injected into the gas chromatograph mod.6890N Network GC System (Agilent Technologies Palo Alto, CA) together in the presence of mass spectrometer mod. 5973 Network Mass Selective Detector (Agilent Technologies Palo Alto, CA) and furnished in the presence of a column HP-5MS (30 m length, 0.25 mm interior diameter, 0.25 um film width Agilent Technologies, Palo Alto, CA). Helium gas was off. Injection was made into a split-splitless injector (split ratio 30:1) at 250°C. The oven program was as follows: 70°C for 3 min, then 6°C/min to 180 for 5 min, then 6°C/min to 280°C for 10 min, then 8°C/min to 290°C for 20 min. The MSD transfer line was set at a temperature of 250°C; MSD temperature quadrupole was of 150°C and ionization temperature was 230°C; mass spectra were seventy electrovolts and scan achievement was accomplished in the series between thirty five and 300m/z. The identification of the components of the* rheum ribes* extract or fraction was assigned by matching their mass spectra with those available in the libraries NIST 02 and WILEY [18].

## 3. Results and Discussion

MCF-7 cell line results have shown that whole plant butanol fraction of* rheum ribes* (WBFRR) exhibited anticancer activity against MCF-7 cell line with IC_50_ value of 36.01± 0.26. Whole plant methanol extract of* rheum ribes* (WMERR), whole plant n-hexane fraction of* rheum ribes* (WHFRR), and whole plant aqueous fraction of* rheum ribes* (WAFRR) were inactive against anticancer (MCF-7) cell line. The anticancer activities (MCF-7 cell line) of extract and fractions of whole plant of* rheum ribes* are shown in Table 1.

Whole plant methanol extract of* rheum ribes* (WMERR), whole plant aqueous fraction of* rheum ribes* (WAFRR), and whole plant butanol fraction of* rheum ribes* (WBFRR) showed anti-inflammatory activity on ROS having IC_50_ value of 23.2±1.9, 24.2±2.7 and 12.0±0.6. The anti-inflammatory activities of extract and fractions of rheum ribes are shown in Table 2.

whole plant butanol fraction of* rheum ribes* (WBFRR) showed Brine shrimp lethality with LD50 693.302 while whole plant methanol extract of* rheum ribes* (WMERR) and whole plant aqueous fraction of* rheum ribes* (WAFRR) showed high lethality at highest concentration. Brine shrimp lethality assay of extract and fractions of rheum ribes is shown in Tables 3–6.

Molecular formula, molecular mass, structure, and RT of compounds 1-21 of whole plant butanol fraction of* rheum ribes* (WBFRR) are shown in Tables 7–10 while mass spectra interpretation of compounds 1-21 of whole plant butanol fraction of* rheum ribes* (LBFRR) is presented in Tables 11–14.

## 4. Conclusion

In this study, GC-MS analysis has shown that whole plant butanol fraction of* rheum ribes* (WBFRR) is composed of 21 compounds which exhibited anticancer (MCF-7) activity having IC_50_ value of 36.01± 0.26. Whole plant methanol extract of* rheum ribes* (WMERR), whole plant n-hexane fraction of* rheum ribes* (WHFRR), and whole plant aqueous fraction of* rheum ribes* (WAFRR) were inactive against anticancer (MCF-7) cell line.

Whole plant methanol extract of* rheum ribes* (WMERR), whole plant aqueous fraction of* rheum ribes* (WAFRR), and whole plant butanol fraction of* rheum ribes* (WBFRR) showed anti-inflammatory activity on ROS having IC_50_ value of 23.2±1.9, 24.2±2.7, and 12.0±0.6.

Whole plant butanol fraction of* rheum ribes* (WBFRR) showed Brine shrimp lethality with LD50 693.302 while whole plant methanol extract of* rheum ribes* (WMERR) and whole plant aqueous fraction of* rheum ribes* (WAFRR) showed high lethality at highest concentration.

This study revealed that whole plant butanol fraction of* rheum ribes* (WBFRR) exhibited significant anticancer MCF-7 activity. In the near future, the constituent of whole plant butanol fraction of* rheum ribes* (WBFRR) can be the alternative drug against MCF-7 cell line with the least toxicity and side effects.

## Figures and Tables

**Table 1:** Anticancer Assay (MCF-7) of extracts and fractions of whole plant of *Rheum ribes*.

S.No.	Extract/Fraction/Std. Drug	Conc. (ug/ml)	% Inhibition/Stimulation	IC50 ± S.D.
1	WMERR	50	38.17	Inactive

2	WHFRR	50	22.46	Inactive

3	WAFRR	50	9.90	Inactive

4	WBFRR	50	93.15	36.01± 0.26

5	Doxorubicin	50	73.23	0.80± 0.05

**Table 2:** Anti-inflammatory activities of extracts and fractions of whole plant of *Rheum ribes*.

S.NO	Extract/Fraction/Std. Drug	Conc. (ug/ml)	% Inhibition/Stimulation	IC50 ± S.D.
1	WMERR	250,50,10	-	23.2± 1.9

2	WHFRR	250,50,10	-	-

3	WAFRR	250,50,10	-	24.2± 2.7

4	WBFRR	250,50,10	-	12.0± 0.6

5	Ibuprofen			11.2± 1.9 ug/ml

**Table 3:** Brine Shrimp Lethality Bioassay of Whole plant Methanol Extract of *Rheum ribes* (WMERR).

Dose (ug/ml)	No. of Shrimps	No. of Survivors	LD50 (ug/ml)	STD. Drug	LD50 (ug/ml)
10	30	28	-	Etoposide	7.4625
100	30	27			
1000	30	00			

**Table 4:** Brine Shrimp Lethality Bioassay of Whole Plant n-Hexane Fraction of *Rheum ribes *(WHFRR).

Dose (ug/ml)	No. of Shrimps	No. of Survivors	LD50 (ug/ml)	STD. Drug	LD50 (ug/ml)
10	30	28	-	Etoposide	7.4625
100	30	24			
1000	30	00			

**Table 5:** Brine Shrimp Lethality Bioassay of Whole Plant Aqueous Fraction of *Rheum ribes* (WAFRR).

Dose (ug/ml)	No. of Shrimps	No. of Survivors	LD50 (ug/ml)	STD. Drug	LD50 (ug/ml)
10	30	29	-	Etoposide	7.4625
100	30	24			
1000	30	00			

**Table 6:** Brine Shrimp Lethality Bioassay of Whole Plant Butanol Fraction of *Rheum ribes* (WBFRR).

Dose (ug/ml)	No. of Shrimps	No. of Survivors	LD50 (ug/ml)	STD. Drug	LD50 (ug/ml)
10	30	30	693.302	Etoposide	7.4625
100	30	29			
1000	30	11			

**Table 7:** Molecular formula, molecular mass, structure, and RT of compounds 1-5 whole plant butanol fraction of *rheum ribes* (WBFRR).

Compound	Name	Molecular Formula	Molecular Mass	Structure	RT
1	1H-Inden-1-one, 2,3-dihydro-3,3,6-trimethyl-	C12H14O	174	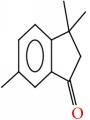	21.411

2	Butanoic acid, 4-(1,1-dimethylethoxy)-3-hydroxy-, methyl ester, (R)-	C9H18O4	190	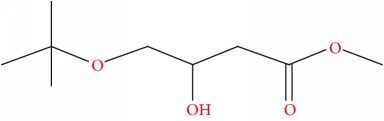	22.644

3	Octaethylene glycol monododecyl ether	C28H58O9	538	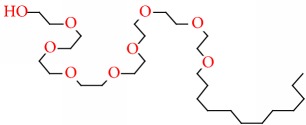	22.804

4	Silane, (diphenylmethoxy)trimethyl-	C16H20OSi	256	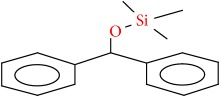	23.140

5	Tetradecanoic acid, 2-hydroxy-	C14H28O3	244	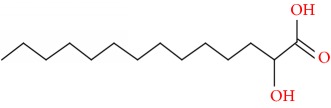	24.015

**Table 8:** Molecular formula, molecular mass, structure, and RT of compounds 6-10 whole plant butanol fraction of *rheum ribes* (WBFRR).

Compound	Name	Molecular Formula	Molecular Mass	Structure	RT
6	Butanedioic acid, dibutyl ester	C12H22O4	230	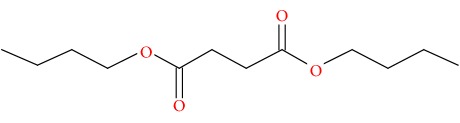	25.151

7	Butanedioic acid, hydroxy-, dibutyl ester, (±)-	C12H22O5	246	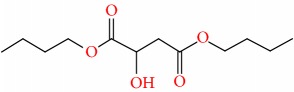	26.902

8	Pipradrol	C18H21NO	267	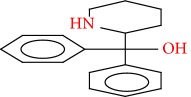	27.076

9	Tetradecanoic acid	C14H28O2	228	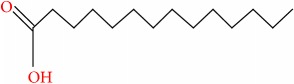	30.280

10	n-Hexadecanoic acid	C16H32O2	256	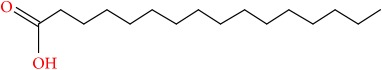	39.870

**Table 9:** Molecular formula, molecular mass, structure, and RT of compounds 11-15 whole plant butanol fraction of *rheum ribes* (WBFRR).

Compound	Name	Molecular Formula	Molecular Mass	Structure	RT
11	Hexanedioic acid, bis(2 methylpropyl) ester	C14H26O4	258	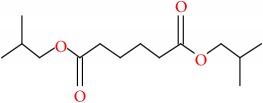	40.231

12	Butyl citrate	C18H32O7	360	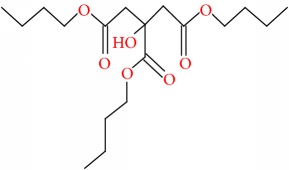	44.843

13	2-Bromotetradecanoic acid	C14H27BrO2	307	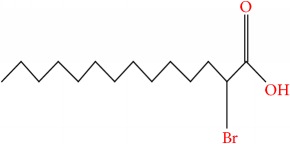	45.437

14	(2,3-Diphenylcyclopropyl)methyl phenyl sulfoxide, trans-	C22H20OS	332	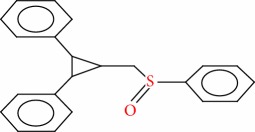	48.124

15	9,10-Secocholesta-5,7,10(19)-triene-3,24,25-triol, (3*β*,5Z,7E)-	C27H44O3	416	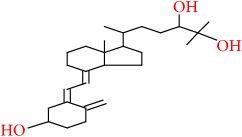	48.653

**Table 10:** Molecular formula, molecular mass, structure, and RT of compounds 16-21 whole plant butanol fraction of *rheum ribes* (WBFRR).

Compound	Name	Molecular Formula	Molecular Mass	Structure	RT
16	Ethyl iso-allocholate	C26H44O5	436	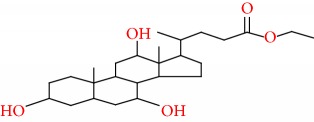	54.708

17	*β*-D-Mannofuranoside, 2,3:5,6-DI-O-ethylboranediyl-1-O-(stigmasta-5, 22-dien-3-yl)-	C39H64B2O6	650	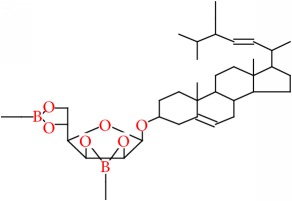	55.215

18	9,19-Cyclolanost-24-en-3-ol, acetate, (3*β*)-	C32H52O2	468	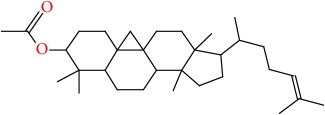	55.985

19	Stigmastan-3,5-diene	C29H48	396	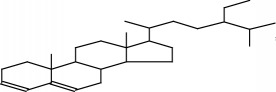	56.278

20	Vitamin E	C29H50O2	430	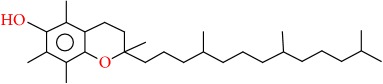	56.655

21	9,19-Cyclolanost-24-en-3-ol, acetate, (3*β*)-	C32H52O2	468	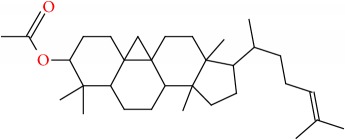	57.338

**Table 11:** Mass spectra of compounds 1-5 of whole plant butanol fraction of *rheum ribes* (LBFRR).

Compound	m/z ( % relative abundance)
1	174(M^+^], 300), 160(125), 159(999), 131(90), 116(110), 115(172), 91(107), 51(86), 39(116), 27(80)

2	190(M^+^), 117(238), 104(188), 103(466), 85(191), 71(167), 59(134), 57(999), 43(454), 41(287), 29(219)

3	538(M^+^), 89(468), 87(229), 73(351), 71(213), 59(182), 57(384), 45(999), 44(232), 43(338), 29(196)

4	256(M+], 378), 241(218), 179(444), 178(240), 168(176), 167(999), 165(400), 152(180), 73(680), 45(244)

5	244(M^+^), 89(999), 71(389), 69(220), 57(285), 55(454), 43(727), 42(233), 41(610), 29(363), 27(194)

**Table 12:** Mass spectra of compounds 6-10 of whole plant butanol fraction of *rheum ribes* (LBFRR).

Compound	m/z ( % relative abundance)
6	230(M^+^), 157(148), 102(45), 101(999), 57(180), 56(159), 55(67), 41(167), 29(133), 28(72), 27(49)

7	246(M^+^), 173(78), 145(304), 117(170), 90(155), 89(999), 71(214), 57(554), 56(143), 41(262), 29(251)

8	267(M^+^), 182(16), 165(24), 152(15), 105(69), 85(50), 84(999), 82(12), 77(73), 56(50), 55(12)

9	228(M^+^), 129(371), 85(241), 73(999), 71(338), 69(282), 60(841), 57(609), 55(451), 43(681), 41(461)

10	256(M^+^), 129(435), 83(267), 73(980), 71(373), 69(351), 60(999), 57(840), 55(767), 43(817), 41(574)

**Table 13:** Mass spectra of compounds 11-15 of whole plant butanol fraction of *rheum ribes* (LBFRR).

Compound	m/z ( % relative abundance)
11	258(M^+^), 185(585), 129(999), 111(264), 101(136), 100(124), 57(463), 56(225), 55(216), 41(216), 29(145)

12	360(M^+^), 259(159), 186(103), 185(999), 129(676), 111(104), 57(180), 56(86), 43(59), 41(200), 29(118)

13	307(M^+^), 99(339), 83(299), 73(436), 69(386), 57(616), 55(712), 43(946), 41(999), 29(473), 27(296)

14	332(M^+^), 207(238), 193(89), 130(103), 129(959), 128(137), 117(83), 115(160), 92(57), 91(999), 77(98)

15	416(M^+^), 136(999), 118(920), 81(655), 69(672), 67(606), 59(841), 57(713), 55(999), 44(999), 43(999)

**Table 14:** Mass spectra of compounds 16-21 of whole plant butanol fraction of *rheum ribes* (LBFRR).

Compound	m/z ( % relative abundance)
16	436(M^+^), 83(460), 81(547), 69(609), 57(797), 55(914), 44(492), 43(999), 41(867), 29(476), 17(469)

17	650(M^+^), 395(282), 394(440), 255(268), 111(255), 99(245), 83(999), 81(256), 69(441), 57(256), 55(430)

18	468(M^+^), 121(278), 109(324), 107(311), 95(456), 93(260), 81(316), 69(752), 55(485), 43(999), 41(572)

19	397(M+1], 165), 396(M^+^], 547), 147(201), 145(157), 105(167), 81(233), 57(269), 55(291), 43(999), 41(209)

20	430(M^+^], 318), 166(117), 165(999), 164(326), 85(135), 71(226), 57(410), 55(185), 43(419), 41(171)

21	468(M^+^), 121(278), 109(324), 107(311), 95(456), 93(260), 81(316), 69(752), 55(485), 43(999), 41(572)

## Data Availability

The data used to support the findings of this study are available from the corresponding author upon request.
